# Lung function in the immediate postoperative period after videoassisted thoracoscopic and thoracotomy pulmonary resection

**DOI:** 10.1186/cc13444

**Published:** 2014-03-17

**Authors:** T Végh, R Nemes, B Fülesdi

**Affiliations:** 1 University of Debrecen, Medical and Health Science Center, Debrecen, Hungary

## Introduction

Previous studies reported that video-assisted thoraco- scopic surgery (VATS) is more beneficial for pulmonary lobectomy concerning the late postoperative period than open thoracotomy [1,2]. However, the early postoperative period when the rate of complications is highest has been scarcely examined. Our study aimed to compare the residual pulmonary function of pulmonary lobectomy patients after VATS and the standard thoracotomy approach in the immediate postoperative period.

## Methods

This prospective study included 14 VATS and 13 thoracotomy lobectomy (THOR) patients (age 58.8 ± 10.9 vs. 59 ± 8.9 years, *P *= 0.96; preoperative FVC 3.4 ± 1 vs. 3.4 ± 0.95 l, *P *= 0.98 and preoperative FEV1 2.7 ± 0.96 vs. 2.7 ± 0.6 l, *P *= 0.9, respectively). All patients received standard surgical and postoperative care with standardized pain management including i.v. diclofenac combined with epidural administration of bupivacaine and fentanyl. Spirometry was performed with a bedside MIR Spirolab II spirometer (Rome, Italy) preoperatively and 4, 8, 24, 48 and 72 hours after the surgery. FVC, fEv1, PaO_2_, PaCO_2_, complication rate, and length of ICU and hospital stay were recorded. Postoperatively measured FVC and FEV1 values were divided by preoperatively predicted values and multiplied by 100 to give the normalized FVC (nFVC) and FEV1 (nFEV1) [3].

## Results

The nFVC and nFEV1 values were significantly higher in the VATS group in the fourth and eighth postoperative hours compared with the THOR group (84.3 ± 14.4 vs. 64.3 ± 23.4, *P *= 0.013 and 84 ± 18.8 vs. 64 ± 22, *P *= 0.017, respectively). There was no statistically significant difference between the groups in the 24th, 48th and 72nd hours, although VATS patients showed higher values at each time points. The length of ICU stay was similarly 1 day, but the length of hospital stay was significantly longer in the THOR group (5 (3 to 6) vs. 7 (3 to 42) days (median and range) (*P *= 0.011)).

## Conclusion

Preoperative lung function prediction severely overestimates the real lung capacity of lobectomy patients in the immediate postoperative period. VATs lobectomy seems more beneficial from the point of early postoperative lung function. VATS lobectomy should be considered in patients with poor preoperative spirometry results to ensure better postoperative outcome.
